# Harzianic acid exerts antimicrobial activity against Gram-positive bacteria and targets the cell membrane

**DOI:** 10.3389/fmicb.2024.1332774

**Published:** 2024-01-29

**Authors:** Xudong Ouyang, Jelmer Hoeksma, Wouter A.G. Beenker, Samantha van der Beek, Jeroen den Hertog

**Affiliations:** ^1^Hubrecht Institute-KNAW and University Medical Center Utrecht, Utrecht, Netherlands; ^2^Institute Biology Leiden, Leiden University, Leiden, Netherlands

**Keywords:** harzianic acid, antimicrobial, antimicrobial resistance, dynamic bacterial morphology imaging (DBMI), mechanism of action

## Abstract

The thermophilic fungus *Oidiodendron flavum* is a saprobe that is commonly isolated from soil. Here, we identified a Gram-positive bacteria-selective antimicrobial secondary metabolite from this fungal species, harzianic acid (HA). Using *Bacillus subtilis* strain 168 combined with dynamic bacterial morphology imaging, we found that HA targeted the cell membrane. To further study the antimicrobial activity of HA, we isolated an HA-resistant strain, *Bacillus subtilis* strain M9015, and discovered that the mutant had more translucent colonies than the wild type strain, showed cross resistance to rifampin, and harbored five mutations in the coding region of four distinct genes. Further analysis of these genes indicated that the mutation in *atpE* might be responsible for the translucency of the colonies, and mutation in *mdtR* for resistance to both HA and rifampin. We conclude that HA is an antimicrobial agent against Gram-positive bacteria that targets the cell membrane.

## 1 Introduction

Fungi interact with their surroundings by production and secretion of biologically active compounds, secondary metabolites (SMs), which are not essential for fungal growth (De Tommaso et al., 2020). These compounds are chemically distinct small molecules often with biological activities that are produced at specific stages of growth to perform important functions, including survival from harsh environments, communication with invaders or alteration of fungal development (Bennett and Bentley, 1989). SMs are synthesized along different pathways than primary metabolites (Vinale et al., 2014) and they are excellent sources of potential therapeutic drugs (Keller, 2019).

The genus of *Oidiodendron* was established under the *Myxotrichaceae* family by Robak (1932). Species of *Oidiodendron* are known as saprobes and are commonly isolated from a wide range of habitats, including soil, decaying plant materials, marine sediments and decomposing human hair (Barron, 1962; Morrall, 1968; Calduch et al., 2004; Roose-Amsaleg et al., 2004). They primarily occur through the temperate regions, with a few exceptions from tropical and subtropical locales. The widespread distribution of this genus is consistent with their excellent adaptive capacity, by which they establish various interactions with other organisms (Couture et al., 1983; Martino et al., 2003; Rice and Currah, 2005). Whereas the genus of *Oidiodendron* has drawn much attention with respect to studies of morphology and taxonomy (Hambleton et al., 1998; Rice and Currah, 2005), the SMs of this genus have not been investigated extensively.

Previously, in order to search for novel bioactive compounds, we have established a fungal SMs library containing more than 10,000 fungal species (Hoeksma et al., 2019), including several *Oidiodendron* strains. One of these strains, *Oidiodendron flavum*, was found to produce an antimicrobial activity, which was identified as harzianic acid (HA) (Ouyang et al., 2021). HA was first isolated as a novel antimicrobial agent from a fungal strain *Trichoderma harzianum* in 1994 (Sawa et al., 1994). Surprisingly, subsequent research focused more on the activity of HA as a plant promotor rather than an antimicrobial (Vinale et al., 2009, 2014). Recently, more interest has been drawn back into its antimicrobial activities. Xie et al. (2021) has described HA as an inhibitor of acetohydroxyacid synthase (AHAS) in fungi. However, in bacteria, although its activity against pathogenic bacteria has been reported (De Filippis et al., 2020), not much data are available on its targets yet. Since HA strongly inhibits bacterial growth, it is suggested to be a promising candidate antibacterial agent. Thus, further research into identification of its mechanism of action (MoA) is required. Here, we determined the antimicrobial spectrum of HA on 14 bacterial strains, and investigated HA’s MoA using dynamic bacterial morphology imaging (DBMI) (Ouyang et al., 2022). In addition, we have developed an HA resistant *Bacillus subtilis* strain by continuous exposure to HA. This strain produced translucent colonies and was four times more resistant to HA. Surprisingly, compared to wild type, this mutant strain was resistant to up to eight times higher concentrations of another antimicrobial agent, rifampin, as well. Genetic analysis of this mutant strain indicated that a recently identified multidrug resistance operon was involved in the enhanced resistance to HA.

## 2 Results

### 2.1 Identification of HA

**FIGURE 1:**
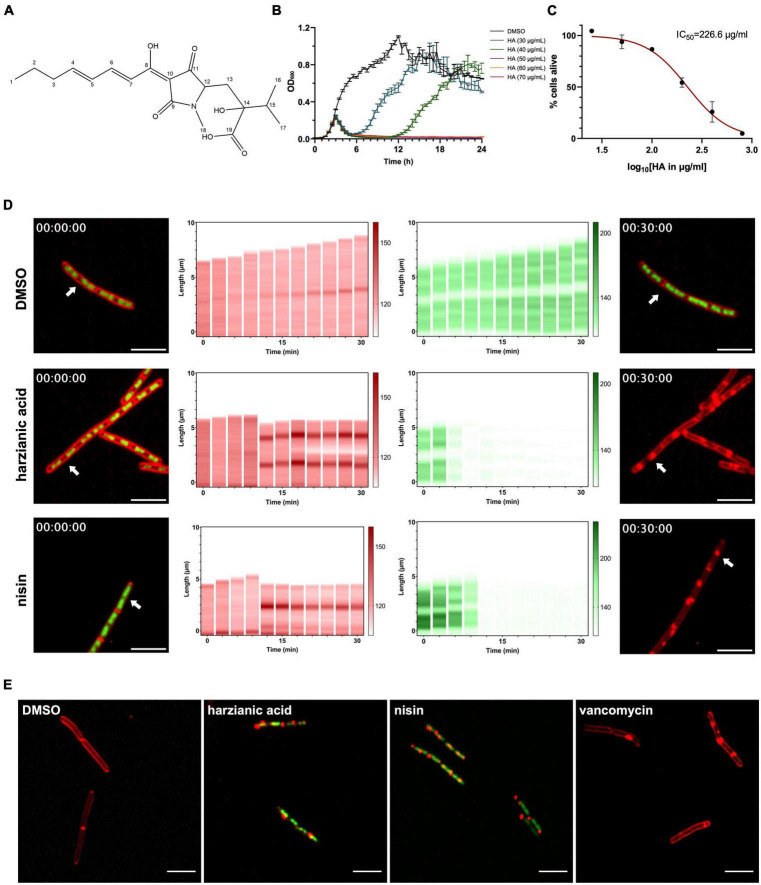
Antimicrobial property of HA. **(A)** Chemical structure of HA. **(B)** Growth curves of *B. subtilis* strain 168/WT in the presence of a range of HA concentrations. OD_600_ was measured every 30 min. HA was added at 2.5 h (arrow). The graph depicts the average and the SEM of biological triplicates. **(C)** Cytotoxicity assay. HepG2 cells were incubated with HA in different concentrations for 20 h before addition of resazurin. The ability to reduce blue resazurin to red resorufin was measured at 540 nm. The average intensity of DMSO control was set as 100% alive and the percentage of intensity from each treated sample was calculated. The mean from biological triplicates was plotted with error bars representing the SEM in black. Non-linear regression was analyzed and plotted in red, on which IC_50_ was based. **(D)** DBMI patterns of cells upon antimicrobial treatments. *B. subtilis* cells were treated with HA (2.5 × MIC), nisin (2.5 × MIC) or 1% DMSO (control). Cells were stained with FM4-64 (red, cell membrane) and SYTO-9 (green, nucleoid) and imaged by time lapse confocal fluorescence microscopy for 30 min with 3 min intervals. The first and last column show the actual micrographs of the first and the last image of the series. The FM4-64 (second column) and SYTO-9 (third column) signals in a single cell (arrow in micrographs) were quantified over the length of the cell and plotted (y-axis, numbers in μm) over time (x-axis, numbers in min). Representative cells are shown. Scale bar is 5 μm. **(E)** Cell permeability assay. *B. subtilis* cells were treated with antimicrobials (2.5 × MIC) or 1% DMSO (control) for 60 min. Cells were stained with FM4-64 (red, cell membrane) and SYTOX-Green (green, nucleoid), and imaged by confocal fluorescence microscopy. Representative images are shown. Scale bar is 5 μm.

In a screen for antimicrobial activity, SMs from *O. flavum* were identified to have potent activity. In order to identify the active compound from *O. flavum*, the agar culture of this fungus was extracted using ethyl acetate and fractionated by high performance liquid chromatography (HPLC). The pure active fraction was then tested for its UV-Vis spectrum, which showed maximum absorbance at 244 and 363 nm. Next, high resolution mass spectrometry (HRMS) of the active compound revealed a mass of the sodium adduct of 388.1750, which suggested several options for a molecular formula. Finally, the remainder of the fraction was dried and used for elemental composition analyses, showing mass percentages of 61.4% carbon, 21.7% oxygen, 7.0% hydrogen, and 3.8% nitrogen. Based on these analyses, we determined a molecular formula of C_19_H_27_NO_6_. Through NMR spectroscopy, we obtained ^1^H, ^13^C, Heteronuclear Single-Quantum Correlation Spectroscopy (HSQC), Heteronuclear Multiple-Bond Correlation spectroscopy (HMBC) and homonuclear correlation (COSY) spectra (Supplementary Figure 1, Supplementary Table 1), which were consistent with data previously reported for HA (Sawa et al., 1994; Vinale et al., 2014; Figure 1A). Thus, we concluded that HA is the antimicrobial compound from *O. flavum*.

Harzianic acid: C_19_H_27_NO_6_, dark yellow powder. HRMS: found 388.1750 (M + Na), calculated 388.1736 for C_19_H_27_NO_6_Na. Elemental composition analyses: C 61.4%; O 21.8%; H 7.0%; N 3.8%. NMR (600 MHz, CDCl_3_): see Supplementary Table 1. UV-Vis λ_*max*_: 244 nm, 363 nm.

### 2.2 HA is an antimicrobial agent against Gram-positive bacteria that affects the cell membrane

Harzianic acid (HA) was tested against a panel of 14 pathogenic bacteria, including seven Gram-positive and seven Gram-negative strains (Supplementary Table 2). The growth of all Gram-positive bacteria was affected with minimum inhibitory concentrations (MICs) ranging from 25 to 200 μg mL^–1^ of HA. The panel included antibiotic-resistant pathogenic bacteria, which were sensitive to HA, e.g., methicillin resistant *Staphylococcus aureus* (MRSA) at 200 μg mL^–1^ and vancomycin resistant *Enterococcus faecium* (VRE) at 100 μg mL^–1^. No inhibition was observed in response to HA of any of the Gram-negative bacteria tested up to 400 μg mL^–1^. This indicates that HA is a selective antimicrobial agent against Gram-positive bacteria.

To further explore the antimicrobial properties of HA against Gram-positive bacteria, a model Gram-positive organism, *B. subtilis* strain 168, was used for the following assays. First, to obtain an accurate MIC, the growth curves of *B. subtilis* were measured in the presence of a range of HA concentrations (Figure 1B). Interestingly, a decrease in OD_600_ was observed 30 min after addition of the compound with all concentrations of HA, even with the lowest concentration we tested, 30 μg mL^–1^. A decrease in OD_600_ was not observed with vehicle control (DMSO). At low concentrations (30 and 40 μg mL^–1^), the OD_600_ increased after a lag period, whereas at higher concentrations (50 μg mL^–1^ and higher) the OD_600_ remained low for at least 18 h, indicating no bacterial growth. These results indicate that the MIC of HA for *B. subtilis* was 50 μg mL^–1^.

To assess the toxicity of this compound on human cells, cytotoxicity assays were done using the HepG2 cell line, originating from human liver. The IC_50_ for HA was 226.6 μg mL^–1^ (Figure 1C), indicating that human cells have a higher tolerance for HA than Gram-positive bacteria.

Next, to begin to identify the MoA of HA, we used an imaging-based method we established previously, DBMI, which is able to classify antimicrobial MoA rapidly and accurately (Ouyang et al., 2022). After imaging of HA-treated cells and control-treated cells (Supplementary Movies 1, 2), DBMI patterns were generated (Figure 1D) and compared to the DBMI pattern induced by known antibiotics (Ouyang et al., 2022). HA induced loss of nucleoid fluorescence at an early stage of treatment. Cell growth was totally inhibited at an early stage as well. This was reminiscent of the initial action of cell membrane-active antimicrobials. Cell length actually appeared to decrease when HA was added, suggesting loss of turgor pressure. Big membrane aggregates were observed when intact cells lost their nucleoid fluorescence, i.e., from 12 min onwards (Figure 1D, Supplementary Movie 2). These effects were similar to the effects of nisin (Ouyang et al., 2022; Figure 1D, Supplementary Movie 3), suggesting that HA might generate pores in the membrane, like nisin. Note the gaps along the membrane from 21 min onwards (Figure 1D, Supplementary Movie 2), indicating that part of the cells disintegrated. Whereas disintegrated membranes were observed in nisin-treated cells by static bacterial imaging (Lamsa et al., 2012), we did not observe disintegrated cells in response to nisin by DBMI (Ouyang et al., 2022; Figure 1D, Supplementary Movie 3). The final disintegrated cell membrane of HA-treated cells showed similarities to the cell membrane of vancomycin-treated cells, but the dynamics suggested that pore formation in the membrane was the initial effect of HA. To investigate this, non-cell permeable SYTOX Green was used to stain nucleoids. Evidently, treatment with HA or nisin, but not vancomycin or vehicle control (DMSO) induced SYTOX Green staining of the nucleoid (Figure 1E). Therefore, we conclude that pore formation is the initial effect of HA on bacterial cells.

### 2.3 Screening for HA-resistant mutants

Knowledge about antimicrobial resistance may provide insight into the underlying MoA of the antimicrobial agent. It is well known that prolonged exposure to increasing concentrations of antimicrobial agents induces resistance. To obtain insight into the MoA of HA, we grew several colonies of *B. subtilis* strain 168 in the presence of 1 × MIC of HA in liquid medium for 3 days. Under these conditions, the liquid culture of three colonies grew and these were transferred sequentially to medium with 2-fold higher concentrations of HA. Over a period of 28 days, one culture, dubbed *B. subtilis* strain M9015, grew successfully in medium with 4 × MIC of HA. Resistance against an antimicrobial may be due to either gene mutation or gene expression adaptation. To distinguish between these possibilities, we cultured the resistant bacteria without HA for ten serial passages. HA resistance persisted in the M9015 strain, suggesting that resistance is caused by mutations in the genome.

**FIGURE 2:**
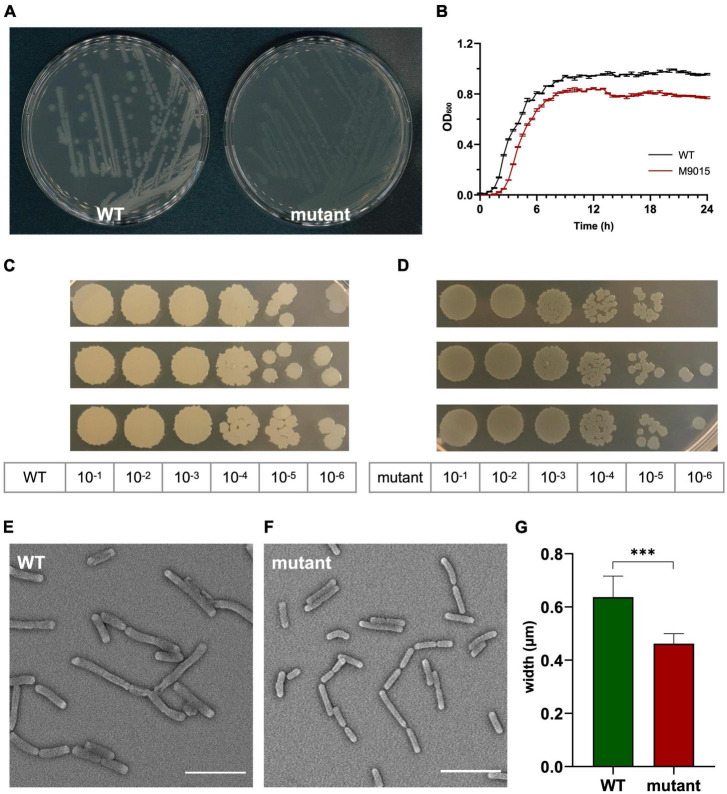
HA-resistant M9015 colonies are translucent and mutant cells are smaller. **(A)** *B. subtilis* strain 168/WT and M9015 plated on LB agar. **(B)** Growth curves of WT and M9015. OD_600_ was measured and the mean from biological triplicates was plotted with error bars representing the SEM. **(C,D)** CFU counting. The overnight cultures (18 h) of WT and M9015 were serially diluted, and 5 μL of each diluted sample was dropped on LB agar. Experiments were performed with biological triplicates. **(E,F)** The cells from overnight cultures (18 h) of WT and M9015 were fixed and imaged by scanning electron microscopy. Representative images are shown. The width of cells was measured and plotted in **(G)**; mean with error bars representing the SEM is depicted. ***indicates *p* ≤ 0.001 by student *t*-text.

The M9015 strain was not only resistant to HA, but the colonies also had a more translucent appearance compared to wild type. However, the colony size did not differ much between the wild type and M9015 strains (Figure 2A). We assumed that this interesting phenotype might be due to slower growth rate (i.e., less cells) or smaller cell size. To investigate this, the growth curves of the strains were first compared. The growth pattern of both strains was similar, but the mutant had a 15 min longer period in the lag phase and a lower final optical density at 600 nm (of ∼0.2 units) in the stationary phase (Figure 2B). Next, the number of cells in the overnight culture was determined (Figures 2C, D). The cell density in the mutant strain was 2.2 × 10^8^ CFU mL^–1^, which was 57% higher than wild type (1.4 × 10^8^ CFU mL^–1^). Comparison of the cell size using a scanning electron microscope (Figures 2E, F) showed that M9015 mutant cells were 27 × 5% smaller than wild type cells (Figure 2G). These results suggest that the cell size affected the appearance of M9015 colonies.

**FIGURE 3:**
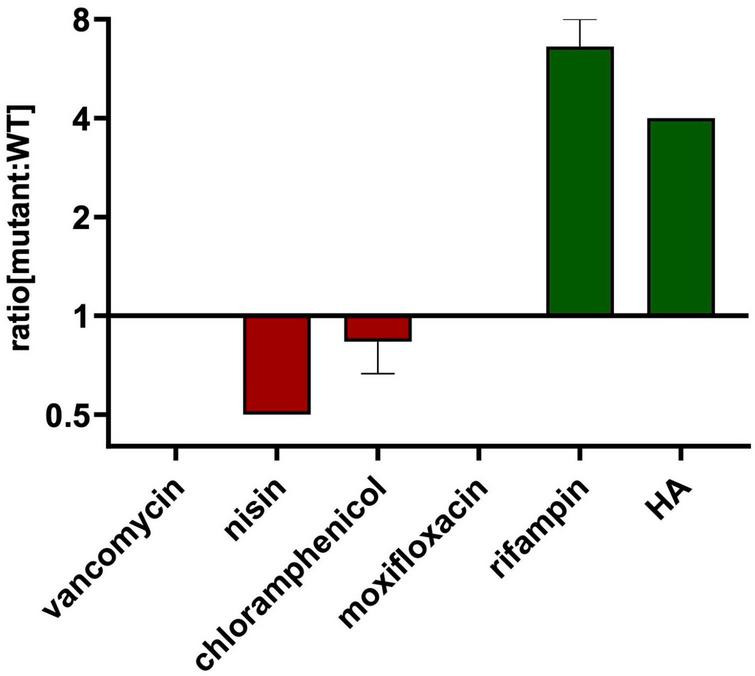
HA-resistant M9015 strain is also resistant to rifampin. MICs of selected antimicrobials against WT or M9015 were measured. The ratio of the MIC of a certain antimicrobial against M9015 to that against WT was calculated. The mean of the ratio from biological triplicates was plotted with error bars representing the SEM. The red bars indicated that M9015 was more sensitive to certain antimicrobial agents whereas the green bars indicated the opposite. The log 2 format was used for the scale of Y axis.

Cross resistance of the M9015 strain to other antimicrobials might provide valuable insight into the antimicrobial class of HA. Therefore, we compared the MICs on M9015 mutant and wild type strains of antimicrobials from five major classes: nisin, vancomycin, chloramphenicol, moxifloxacin and rifampin. Interestingly, M9015 showed reduced sensitivity to rifampin, an RNA class antimicrobial, to an even higher extent than HA (4–8-fold; Figure 3). No significant differences were observed for any of the other antimicrobials, which included antimicrobials targeting the cell envelope. It is surprising that the HA-resistant M9015 strain is more resistant to the RNA class antimicrobial rifampin, because our earlier experiments suggested that HA targets the cell envelope.

### 2.4 Identification of mutations in M9015 by genome sequencing

In order to determine which pathway(s) was/were affected in M9015, the genomes of M9015 mutant and wild type strains were sequenced by Next Generation Sequencing. The published genome sequence of *Bacillus subtilis* subsp. *subtilis* str. 168 was used as the reference genome. The mapping of the reads from our wild type strain against this referenced genome showed high coverage (only around 2,000 mutation sites), suggesting this reference is applicable, which facilitated the alignment of our sequencing data. Bioinformatic comparison of the wild type and mutant genomes resulted in the identification of ten potential variations (Supplementary Table 3), five of which were suggested to be reliable by the Integrative Genomics Viewer (Supplementary Figures 2, 3). These five mutations were located in the coding region of four different genes, *ymaB*, *flgL, atpE*, and *mdtR* (two mutations; *mdtR* was formerly known as *yusO*).

**FIGURE 4:**
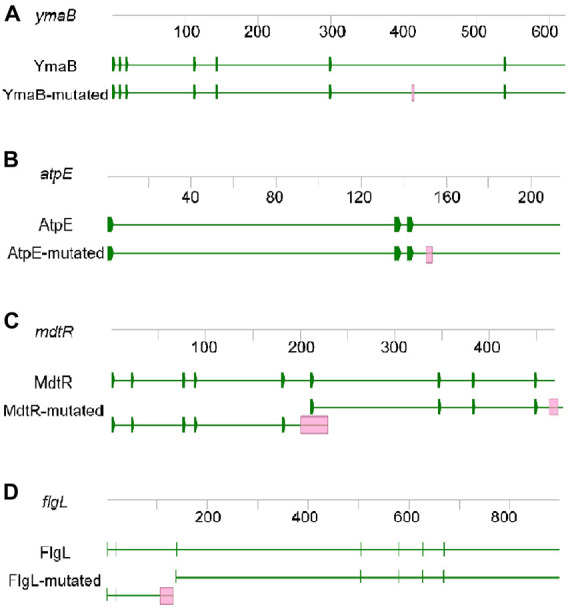
Mutated genes of M9015. The genes of *ymaB* **(A)**, *atpE* **(B)**, *mdtR* **(C)**, and *flgL* **(D)** are shown. Each gene was presented with DNA sequence (grey line with numbers indicating the length of DNA) and protein sequence (green line with green blocks indicating the sites of initiation codon). Red blocks in the mutated gene and protein sequences indicate the mutated sites.

The mutations in *ymaB* and *atpE* were single-base substitutions causing an amino acid substitution in their protein products. YmaB is a putative Nudix hydrolase with RNA pyrophosphohydrolase activity (Frindert et al., 2019), and the mutation we found resulted in a p.L138P substitution (Figure 4A). Although both these amino acids are non-polar and hydrophobic, their structures differ substantially, which might affect the activity of YmaB. The function of this gene is not clearly described in literature yet, and therefore the effect of the observed mutation remains unclear. The *atpE* gene encodes the subunit c of ATP synthase (Walker, 2013). In M9015, we identified a p.A51V substitution in AtpE (Figure 4B). InterPro^1^ predicted the ATP binding site of AtpE to be from p.33A to p.54E, indicating this mutation may be at the binding site. The properties of Ala and Val are quite similar, but the larger size of Val in the p.A51V mutant might affect the activity of AtpE severely.

The *mdtR* gene expresses MdtR, a transcription repressor, which binds to the *mdtR-mdtP* operon promoter region and thus represses *mdtR-mdtP* expression. MdtP was reported to confer low-level resistance to fusidic acid, novobiocin, streptomycin, and actinomycin (Kim et al., 2009). This gene was disrupted by two insertions in M9015 mutant cells: a single-base insertion (c.199_200insA), introducing a frame shift and stop-codon and an in-frame nine-base insertion (c.454_455insGAGGAAACG). Therefore, the gene product would be different from the wild type strain (Figure 4C). Instead of expressing MdtR, the M9015 mutant might produce two (non-functional) truncated proteins from this gene, dubbed MdtR-m1 and MdtR-m2. In MdtR-m1, the frame shift would result in a premature stop p.A67D fsX10. There is an alternate initiation site just past the single base pair insertion in *mdtR*, which might initiate the expression of MdtR-m2. The nine base pair insertion near the 3’ end of *mdtR* would lead to a three amino acid duplication near the C-terminus of MdtR-m2 (p.G153_G155dup). However, whether MdtR-m1 and/or MdtR-m2 proteins are stably expressed in M9015 remains to be determined. Hence, it is not unlikely that mutation of *mdtR* might contribute to HA resistance.

The *flgL* gene expresses FlgL, a flagellar hook-filament junction protein, has a function in motility and chemotaxis (Chan et al., 2014). It contained a one base pair deletion in M9015 cells, resulting in a truncated FlgL protein (p.K35SfsX9, FlgL-m1, Figure 4D). Potentially, a second FlgL protein, FlgL-m2 starting from Met47 of FlgL was produced in M9015 cells as well (Figure 4D). It has been suggested that disruption of this gene may lead to reduced motility, which might contribute to the translucent appearance of M9015 colonies as well.

To further study these gene functions, we ordered four mutants from Bacillus Genetic Stock Center (Koo et al., 2017), each containing a deletion of one of the four indicated genes (Table 1). The colonies of most mutants appeared normal, except for the Δ*atpE* strain, which had smaller and more translucent colonies (Supplementary Figure 4). This indicates that the mutation in *atpE* is responsible for the translucent phenotype.

**TABLE 1:** Minimum inhibitory concentrations (MICs) of HA and rifampin on *B. subtilis* and mutants.

Strain	Source	MIC to HA (μ g mL^–1^)	MIC to rifampin (μ g mL^–1^)
*Bacillus subtilis* strain 168	Laboratory stock	50	0.13
*Bacillus subtilis* strain M9015	This work	200	0.8
*Bacillus subtilis* strain Δ*atpE*	BKK36860*	25	0.13
*Bacillus subtilis* strain Δ*ymaB*	BKK17400*	50	0.13
*Bacillus subtilis* strain Δ*mdtR*	BKK32870*	200	0.66
*Bacillus subtilis* strain Δ*flgL*	BKK35400*	50	0.13
*Bacillus subtilis* strain Δ*mdtP*	BKK32880*	25	0.08

MICs were determined by serial dilution of HA or Rifampin using the indicated strain. The result of biological triplicates is depicted here.

*Strains from Bacillus Genetic Stock Center.

Next, we tested the MICs of HA and rifampin on these four mutants. As listed in Table 1, Δ*mdtR* strain had a similar level of resistance to both HA and rifampin as our M9015 strain. In comparison, none of the other strains showed more resistance to either of the antimicrobials. This suggested that the disruption of *mdtR* is the reason for HA resistance in the M9015 strain. *MdtR* belongs to the *mdtR-mdtP* operon. MdtR is a transcriptional repressor of *mdtP*. MdtR represses expression of MdtP, a multidrug efflux transporter, which confers multidrug resistance (Kim et al., 2009). Loss of function of MdtR results in elevated expression of MdtP and hence resistance to HA. We hypothesized that loss of *mdtP* would lead to hypersensitivity to HA. To test this hypothesis, we tested a *B. subtilis* strain with Δ*mdtP* mutation and assessed the MIC of HA and rifampin. The results (Table 1) showed that this strain is two times more sensitive to both HA and rifampin than the wild type, which is consistent with loss of MdtR function resulting in resistance to HA and rifampin.

### 2.5 HA generated pores in the cell membrane

**FIGURE 5:**
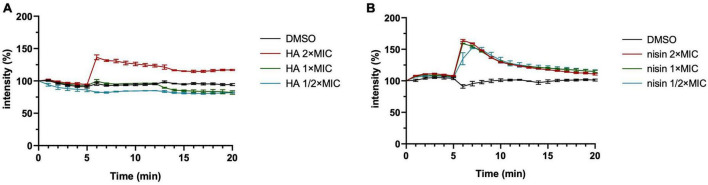
Harzianic acid (HA) affects cell depolarization only at high concentration. *B. subtilis* membrane potential levels were quantified using the fluorescent dye DiSC_3_(5). Different concentrations of HA **(A)** or nisin **(B)** were added after 5 min. DMSO was used as control. The fluorescence was depicted as percentage of the value at the start (*t* = 0 min) (y-axis) over time (x-axis, min). The mean from biological triplicates was plotted with error bars representing the SEM.

We observed effects of HA on the cell envelope (Figures 1D, E), but how HA affects the cell envelope remains to be determined. HA might affect membrane integrity without direct targeting of the membrane. This is not unprecedented, because gentamicin exclusively targets ribosomes, which results in misfolded membrane proteins. Eventually, this leads to membrane defects (Davis et al., 1986; Kohanski et al., 2008). However, gentamycin did not induce changes in the membrane potential under conditions that membrane defects were induced, i.e., treatment with gentamicin for up to 15 min (Supplementary Figure 5). Treatment with HA did affect the membrane potential (Figure 5A), suggesting that it is unlikely that HA affects pore formation by a similar mechanism as gentamycin.

Low concentrations of HA (1 × MIC or 0.5 × MIC) did not affect the membrane potential (Figure 5A), but did inhibit bacterial growth (Figure 1B). Interestingly, nisin treatment showed effects on membrane polarization at all concentrations tested, even at 0.5 × MIC (Figure 5B). We cannot exclude the possibility that HA has multiple targets in a dose-dependent manner, like triclosan. Triclosan is a multi-target antimicrobial that has specific targets in the membrane only at high concentrations (Russell, 2004).

To investigate if *mdtR* was required for HA-induced cell envelope damage, we tested cell permeability of mutant strains M9015 and Δ*mdtR* with HA concentration of 100 μg mL^–1^, which is 1/2 × MIC for these two strains, but 2 × MIC on the wild type strain. As shown in Supplementary Figure 6, pores were generated in both of the mutants, like in the WT control strain. Apparently, functional MdtR was not required for HA-induced pore formation in Gram-positive bacteria.

## 3 Discussion

Here, we describe an initial characterization of the MoA of the antimicrobial agent, HA. The minimum concentration of HA to inhibit *B. subtilis* growth overnight was 50 μg mL^–1^, which was around 4 to 5 times less than the IC_50_ of HA for human cells (Figures 1B, C). Treatment of bacteria with HA at concentrations below 50 μg mL^–1^ also led to rapid arrest of bacterial growth and even lysis of the cells (a reduction in OD_600_). Interestingly, following treatment with 30 or 40 μg mL^–1^ HA, bacterial growth recovered 3–9 h after the start of treatment, respectively, (Figure 1B). The mechanism underlying recovery of the bacterial growth following treatment with HA below 50 μg mL^–1^ remains to be determined. The cells showed delayed but similar growth kinetics to control samples. HA may be degraded by the few live cells, or HA may be exhausted or quenched by the bacterial cells. The remaining live cells appear not to be affected by the previous presence of HA in the medium and are now able to grow freely again at the same growth rate as untreated cells.

Our results suggested that HA is an antimicrobial agent that generated pores in the cell membrane of Gram-positive bacteria. HA did not bind to either of the provided lipids in our study (Supplementary Table 4). Therefore, the mechanism of the pore formation is still unclear. To further study the mechanism, other approaches, e.g., molecular dynamics simulations to model pore formation in lipid bilayers (Tieleman et al., 2003; Tilley and Saibil, 2006), might provide insight into the underlying mechanism.

To further study the MoA of HA, we selected a low-level HA-resistant strain, *B. subtilis* strain M9015, by continuous exposure to HA. The M9015 strain contains four mutated genes compared to wild type. Interestingly, the M9015 strain has more translucent colonies than wild type. We examined this phenotype in relation to HA-resistance. In the comparison of the growth patterns and cell size between mutant and wild type, we found that the size of M9015 cells was reduced. Smaller cell size might contribute to the translucent appearance of colonies, because the colonies will be thinner when the cells are smaller. The observed apparent reduction in growth might also be caused by reduced cell size, because the same number of bacterial cells will have a lower OD_600_ when the cells are smaller. The translucent phenotype of the M9015 strain was similar in the Δ*atpE* strain, which lacks one of the four genes that are mutated in M9015. Given the similarity in phenotype, it is likely that the translucency is caused by lack of *atpE.* Loss of *atpE* is not involved in resistance to HA, because the Δ*atpE* strain showed similar sensitivity to HA as WT (Table 1). One of the four genes, *mdtR*, confers similar resistance to bacteria as observed in the M9015 strain (Table 1). Interestingly, *mdtR* has been implicated in antimicrobial resistance before (Kim et al., 2009). Yet, it has been reported that several point-mutations in *mdtR* did not confer resistance to rifampin (Kim et al., 2009), which is not in line with our results. We found that both the HA-resistant strain M9015 and the Δ*mdtR* strain had cross resistance to rifampin. In the previous work, only part of the gene was knocked out (Kim et al., 2009), which may explain the discrepancy with our results, because in the M9015 strain and the Δ*mdtR* strain, the function of the *mdtR* gene is completely disrupted. Our conclusion that MdtR is involved in resistance is supported by the observation that a *mdtP* mutant is more sensitive to HA (Table 1). *MdtP* is the target of the transcriptional repressor MdtR and encodes a multidrug efflux transporter. It is interesting to note that the M9015 strain was resistant to HA and rifampin, but not vancomycin, nisin, chloramphenicol or moxifloxacin (Figure 3), suggesting that the MdtP multidrug efflux transporter was selective for HA and rifampin.

Recently, HA was found to target acetohydroxyacid synthase (AHAS) in fungi (Xie et al., 2021). AHAS is the first enzyme in the branched-chain amino acid biosynthetic pathway. To determine if bacterial AHAS is the intracellular target of HA, we tested six mutants of *B. subtilis*, each with a mutation in an AHAS related gene, and assessed their sensitivity for HA. None of these mutants had a different MIC than wild type *B. subtilis* (Supplementary Table 5). Multiple genes encode proteins with AHAS catalytic activity in the bacterial genome (Kunst et al., 1997). Due to this redundancy, the HA-sensitivity of mutant bacteria lacking the function of single AHAS encoding genes may not be affected. Alternatively, due to poor sequence conservation, HA may not target any of the AHAS-encoding bacterial genes. Whether inhibition of AHAS contributes to antimicrobial activity of HA toward bacteria remains to be determined.

To conclude, our results suggest that HA is an antimicrobial agent against Gram-positive bacteria, which targets the cell membrane. We have developed a HA-resistant strain, M9015, and discovered that disruption of *mdtR*, a component of the *mdtR-mdtP* multidrug resistance operon was responsible for resistance to HA and rifampin, but not other antimicrobials. Whereas AHAS, the first enzyme in the branched-chain amino acid biosynthetic pathway, appears to be a good candidate, the intracellular target of HA remains to be determined.

## 4 Materials and methods

### 4.1 Strains and reagents

*B. subtilis* strain 168 was used for MoA identification in this study (Kunst et al., 1997). *O. flavum* (CBS 366.71) was obtained from the Westerdijk Fungal Biodiversity Institute (The Netherlands) and used for biologically active compound production. Pathogenic bacterial strains used for activity tests were either obtained from ATCC or they were clinical isolates (kind gift from University Medical Center Utrecht, The Netherlands) and they are listed in Supplementary Table 2. *B. subtilis* mutants were obtained from Bacillus Genetic Stock Center (Koo et al., 2017; Table 1, Supplementary Table 5). Commercial antimicrobials and resazurin were purchased from Sigma Aldrich. FM4-64, DiSC_3_(5) and SYTOX-Green were purchased from Thermo Fisher Scientific.

### 4.2 Identification of HA

Identification of fungal SMs were performed as described before with minor modifications (Hoeksma et al., 2019; Ouyang et al., 2021). *O. flavum* was cultured on Malt Extract Agar (MEA) for 14 days. Secondary metabolites were extracted using ethyl acetate and separated using a Shimadzu preparative high performance liquid chromatography (HPLC) system with a C18 reversed phase Reprosil column (10 μm, 120 Å, 250 × 22 mm). The mobile phase was 0.1% trifluoroacetic acid in water (buffer A) and 0.1% trifluoroacetic acid in acetonitrile (buffer B). A linear gradient was applied of buffer B (5–95%) for 40 min. Fractions were collected and tested on *B. subtilis.* The active fraction was assessed for its purity through Shimadzu LC-2030 analytical HPLC using a Shimadzu Shim-pack GISTC18-HP reversed phase column (3 μm, 4.6 × 100 mm). LC-MS was performed on a Shimadzu LC-system connected to a Bruker Daltonics μTOF-Q mass spectrometer. High resolution mass spectrometry (HRMS) was measured on an LCT instrument (Micromass Ltd, Manchester, UK). Elemental composition analyses were performed by Mikroanalytisch Labor Kolbe (Oberhausen, Germany). Finally, the compound was dissolved in 400 μL CDCl_3_ + 0,03% TMS and analyzed by Nuclear Magnetic Resonance (NMR) spectroscopy. More specifically, ^1^H-NMR, Heteronuclear Single Quantum Coherence (HSQC), Heteronuclear Multiple-Bond Correlation (HMBC), and Correlation spectroscopy (COSY) spectra were measured at 600 MHz using a Bruker instrument. ^13^C-NMR was measured on the same instrument at 150 MHz.

### 4.3 Microdilution assay

Minimum Inhibitory Concentration (MIC) was determined by broth microdilution assay as previously described (Zgoda and Porter, 2001). The freshly prepared early exponential-phase cell cultures of different strains were diluted 1: 100 into Luria-Bertani (LB) medium, and then distributed in a 96-wells plate. Antimicrobials were tested starting at a 10 × dilution of the stock in DMSO, which was then serially diluted with a factor 2. MIC was defined as the lowest dilution at which bacteria did not grow, based on visual inspection after an overnight incubation at 37°C.

### 4.4 Cytotoxicity assay

For the cytotoxicity assay, HepG2 cells were seeded in 96-well plates and grown in DMEM low glucose medium (ThermoFisher, 10567014) supplemented with 10% FBS. Test compounds were added in different concentrations, with a final concentration of 1% DMSO and cells were incubated for 20 h at 37°C with 5% CO_2_. Next, resazurin was added to reach a final concentration of 0.1 mM. After 3 h incubation, the fluorescence was measured on a PHERAstar microplate reader (BMG Labtech) using an excitation wavelength of 540 nm and emission wavelength of 590 nm. The average intensity of DMSO control was set as 100% alive and the percentage of intensity from each treated sample was calculated. IC_50_ was calculated using non-linear regression in GraphPad Prism. Experiments were conducted in biological triplicates.

### 4.5 Growth curves

Overnight bacterial cultures were diluted 1: 50 into fresh LB medium and incubated at 37°C with shaking. OD_600_ of cultures were measured by a FLUOstar microplate reader (BMG Labtech) every 30 min for 24 h.

### 4.6 Confocal microscopy

Microscopy was performed following the protocol described before with minor modification (Müller et al., 2016; Ouyang et al., 2021). Briefly, bacterial cells were treated with antimicrobials (2.5 × MIC) or 1% dimethyl sulfoxide (DMSO) as control for up to 60 min. Samples were then stained with 1.5 μM FM4-64 and 0.5 μM SYTOX-Green, immobilized on microscope slides covered with an agarose pad containing 1% agarose and LB medium, and imaged. Confocal microscopy was carried out using a Perkin Elmer UltraView VoX spinning disk microscope system. Experiments were done with biological triplicates. Images were analyzed using Fiji (Schindelin et al., 2012).

### 4.7 Time-lapse microscopy and DBMI patterns

Time-lapse imaging and data analysis were performed as previously described (Ouyang et al., 2022). Briefly, time-lapse images were taken using a syringe-made single-well agarose pad and the spinning disk microscope system described above for 45 min with 3 min intervals. 400 μL LB medium with HA (2.5 × MIC) or 1% DMSO (control) was added into the well on the agarose pad after the second image was taken. Images were analyzed using Fiji. A wide line (width: 20-pixel, i.e., 1.3 μm) was drawn to cover a whole cell and the intensity of both membrane signal and nucleoid signal over the line was acquired with the tool Plot Profile. The graphs of Plot Profile from each series of time-lapse imaging were then re-plotted into one heatmap with GraphPad Prism to generate the DBMI patterns.

### 4.8 Cell depolarization assay

DiSC_3_(5) is commonly applied for cell depolarization assay because it generally accumulates in well-energized cells. Disruption of membrane potential releases this probe from cells into the medium, resulting in an increase of overall fluorescence in the cell suspension (Kepplinger et al., 2018). To perform this assay, the freshly prepared early exponential-phase cell cultures with an OD_600_ of 0.3 in LB medium were stained with DiSC_3_(5) for 10 min. Then the fluorescence was measured on a PHERAstar microplate reader (BMG Labtech) using a 540 nm/590 nm filter. Experiments were conducted in biological triplicates.

### 4.9 Lipid assay

This assay was applied as previously described with modifications (Kleijn et al., 2016). The appropriate amounts of antagonists lipid I, lipid II and C_55_-P in CHCI_3_: MeOH (1: 1) were added to the 96-well plates and the solvent was evaporated. The antagonists were re-dissolved in LB containing antimicrobials (2 × and/or 1 × MIC) or 1% DMSO in solution. The freshly prepared early exponential-phase cell cultures of *B. subtilis* were diluted 1: 100 into LB medium, and then distributed in the 96-wells plate with antagonists and antimicrobials. After incubation overnight at 37°C, bacterial growth was inspected visually.

### 4.10 Screening for HA-resistant mutants

To obtain HA-resistant mutants, we grew *B. subtilis* in the presence of 1 × MIC of HA in LB medium at 37°C with shaking. Once bacterial growth was observed, it was sequentially transferred to medium with 2-fold higher concentrations of HA. The whole process was set for a period of 28 days.

### 4.11 Scanning electron microscopy

Overnight cultures of *B. subtilis* strain 168 and M9015 were transferred to fresh LB medium and grown to mid-log phase. Bacterial cells were then washed, fixed, dehydrated, mounted onto 12.5 mm specimen stubs and coated with gold to 1 nm as previously described (van der Beek et al., 2015). Samples were imaged using a Phenom PRO desktop SEM (Phenom-World BV). Cell width was measured from 10 cells for each strain from triplicate images using Fiji.

### 4.12 Genomic DNA sequencing

The bacterial cell wall of *B. subtilis* was lysed using 0.5 mg mL^–1^ lysozyme for 1 h at 37°C. Next, genomic DNA was isolated using the Wizard Genomic DNA purification Kit (Promega) and sequenced by Illumina Next Generation Sequencing in University Medical Center Utrecht.

### 4.13 Bioinformatic analysis

The raw sequencing reads were subjected to a panel of bioinformatic analyses including quality control using FastQC and Trimmomatic, alignment to reference genome using BWA-MEM algorithm and variant calling using Integrative Genomics Viewer (IGV). The published genome sequence of *Bacillus subtilis* subsp. *subtilis* str. 168 was used as the reference genome, which has a NCBI accession number of NC_000964.3.

## Data availability statement

The raw data supporting the conclusions of this article will be made available by the authors, without undue reservation.

## Author contributions

XO: Conceptualization, Data curation, Formal Analysis, Investigation, Methodology, Visualization, Writing – original draft, Writing – review & editing. JH: Formal Analysis, Investigation, Methodology, Visualization, Writing – review & editing. WB: Formal Analysis, Investigation, Methodology, Visualization, Writing – review & editing. SvdB: Formal Analysis, Investigation, Methodology, Visualization, Writing – review & editing. JdH: Conceptualization, Funding acquisition, Project administration, Supervision, Visualization, Writing – original draft, Writing – review & editing.

## Footnotes

1
https://www.ebi.ac.uk/interpro/
