# Supplementary material for: Harzianic acid exerts antimicrobial activity against Gram-positive bacteria and targets the cell membrane
Source: Front Microbiol. 2024 Jan 29;15:1332774. doi: 10.3389/fmicb.2024.1332774 (PMC10860749; doi:10.3389/fmicb.2024.1332774)
Supplement: Supplementary file 4 [file Data_Sheet_1.PDF]

Supplementary Material

1 Supplementary Figures and Tables

1.1 Supplementary Figures

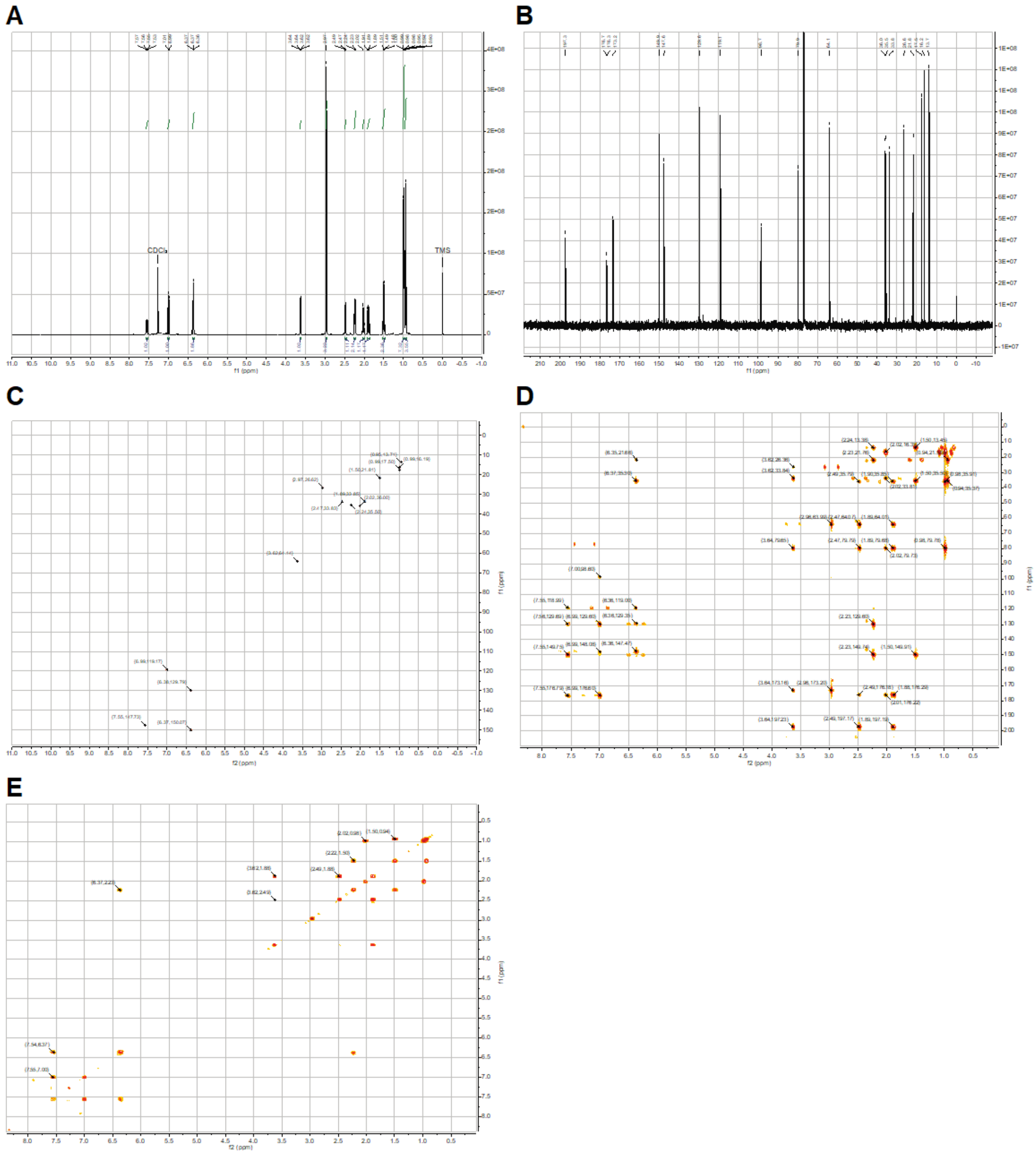

Supplementary Figure 1. NMR spectroscopy of harzianic acid. (A) ^{1}H-NMR spectrum, 600 MHz, $CDCl_3$. (B) ^{13}C-NMR spectrum, 150 MHz, $CDCl_3$. (C) HSQC spectrum, 600 MHz, $CDCl_3$. (D) HMBC spectrum, 600 MHz, $CDCl_3$. (E) COSY spectrum, 600 MHz, $CDCl_3$.

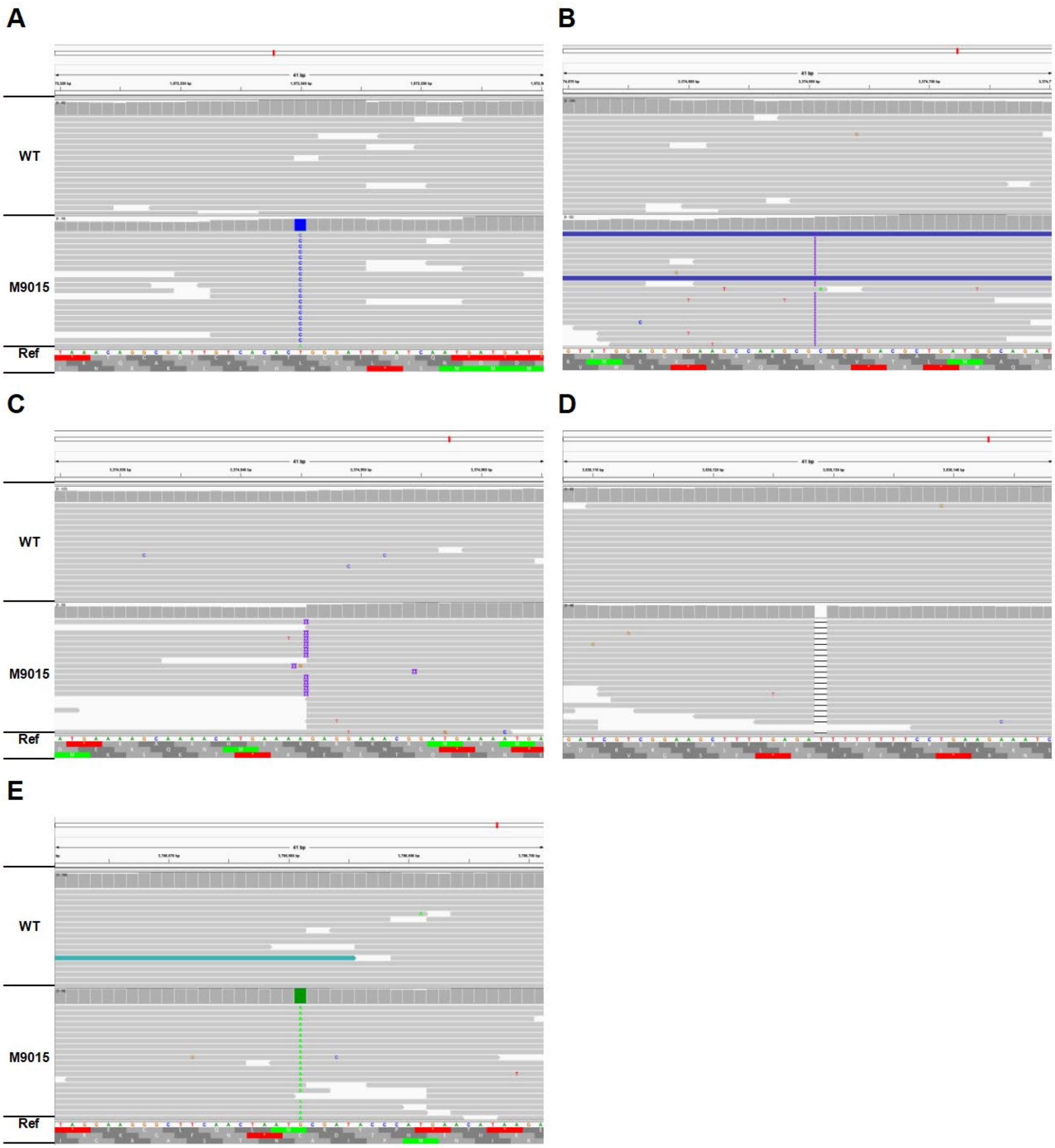

Supplementary Figure 2. Viewing gene variations with Integrated Genomics Viewer (I). 10 predicted mutated sites were verified using Integrated Genomics Viewer. Five correctly predicted mutations (Mutation#2, #7-#10) (see Table S3) were listed in (A) to (E). "Ref" indicated the reference genome. Representative reads (around 15) were presented for both WT and M9015.

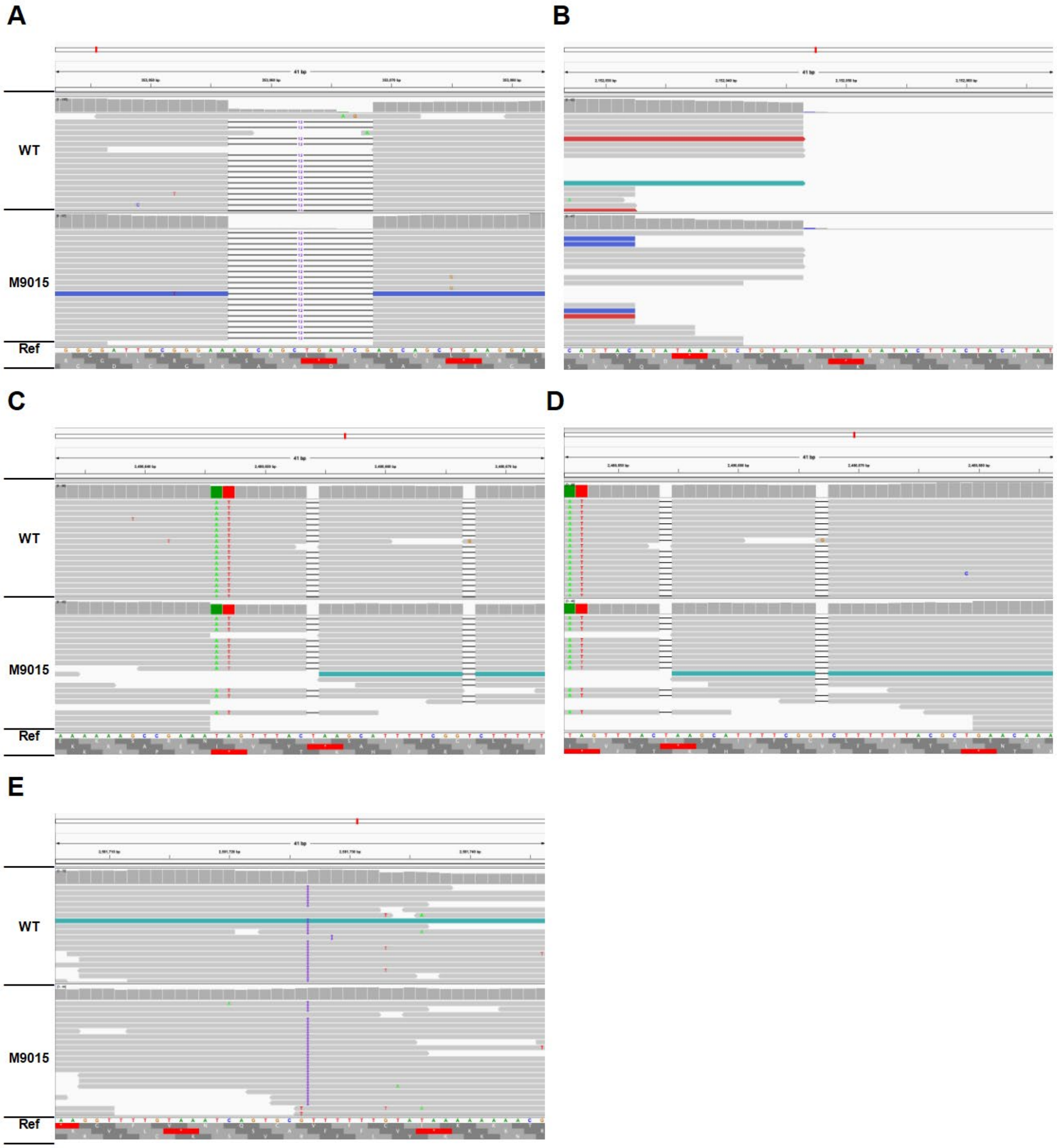

Supplementary Figure 3. Viewing gene variations with Integrated Genomics Viewer (II). 10 predicted mutated sites were verified using Integrated Genomics Viewer. Five wrongly predicted mutations (Mutation#1, #3-#6) (see Table S3) were listed in (A) to (E). “Ref” indicated the reference genome. Representative reads (around 15) were presented for both WT and M9015.

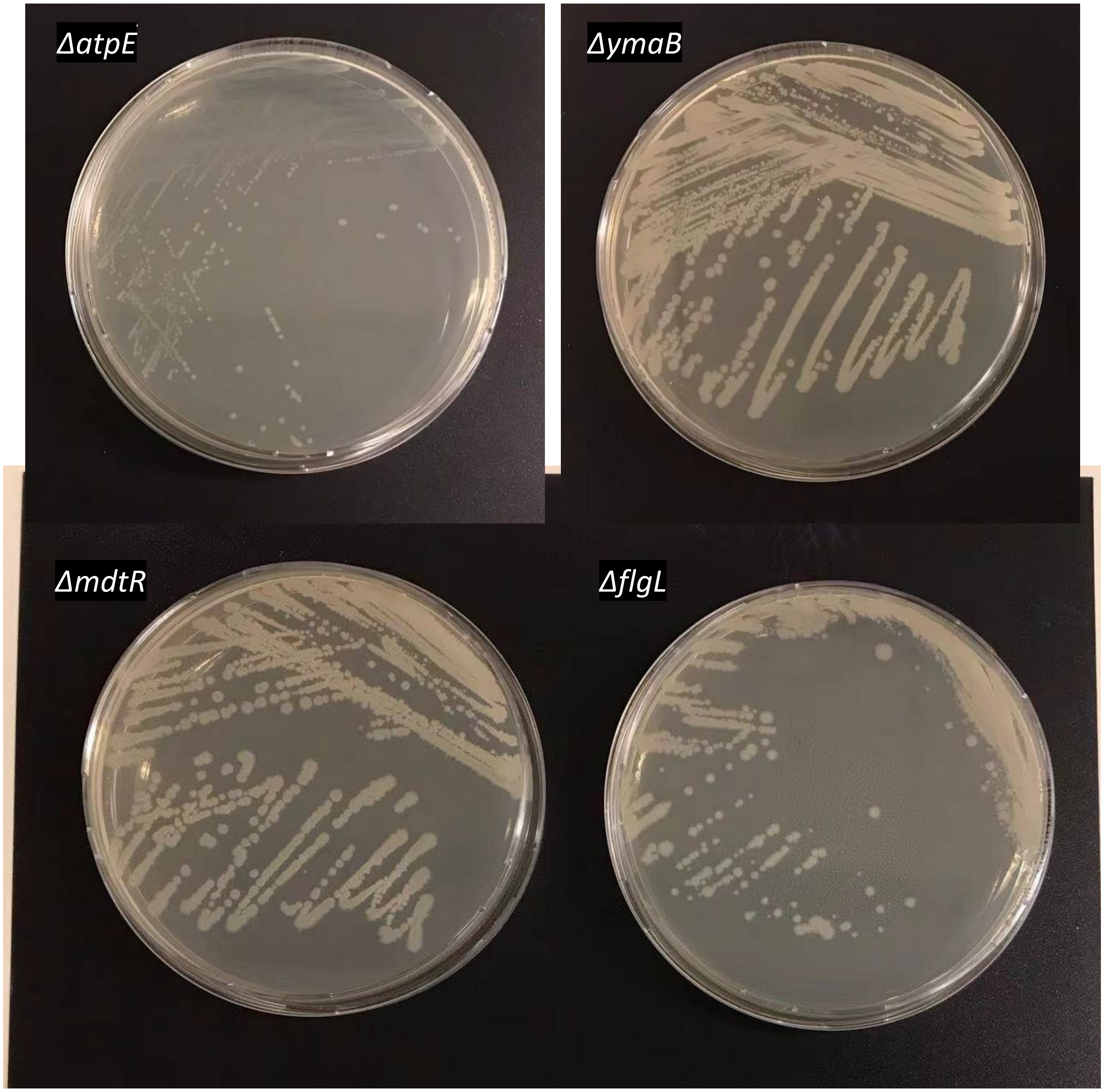

Supplementary Figure 4. Colony appearance of four mutants of *B. subtilis*. *B. Subtilis* mutants were cultured on LB agar at 37 °C overnight. Plates were imaged.

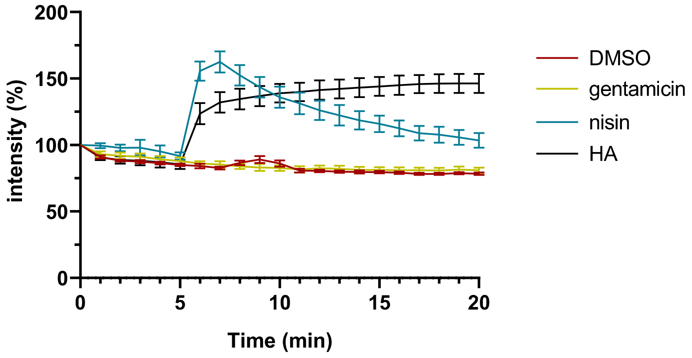

Supplementary Figure 5. Gentamicin did not affect cell depolarization. *B. subtilis* membrane potential levels were quantified using the fluorescent dye DiSC$_3$(5). Gentamicin (5 × MIC), HA (5 × MIC), DMSO (blank control) or nisin (positive control, 5 × MIC) were added after 5 min. The fluorescence was depicted as percentage of the value at the start (t = 0min) (y-axis) over time (x-axis, min). The mean from biological triplicates was plotted with error bars representing the SEM.

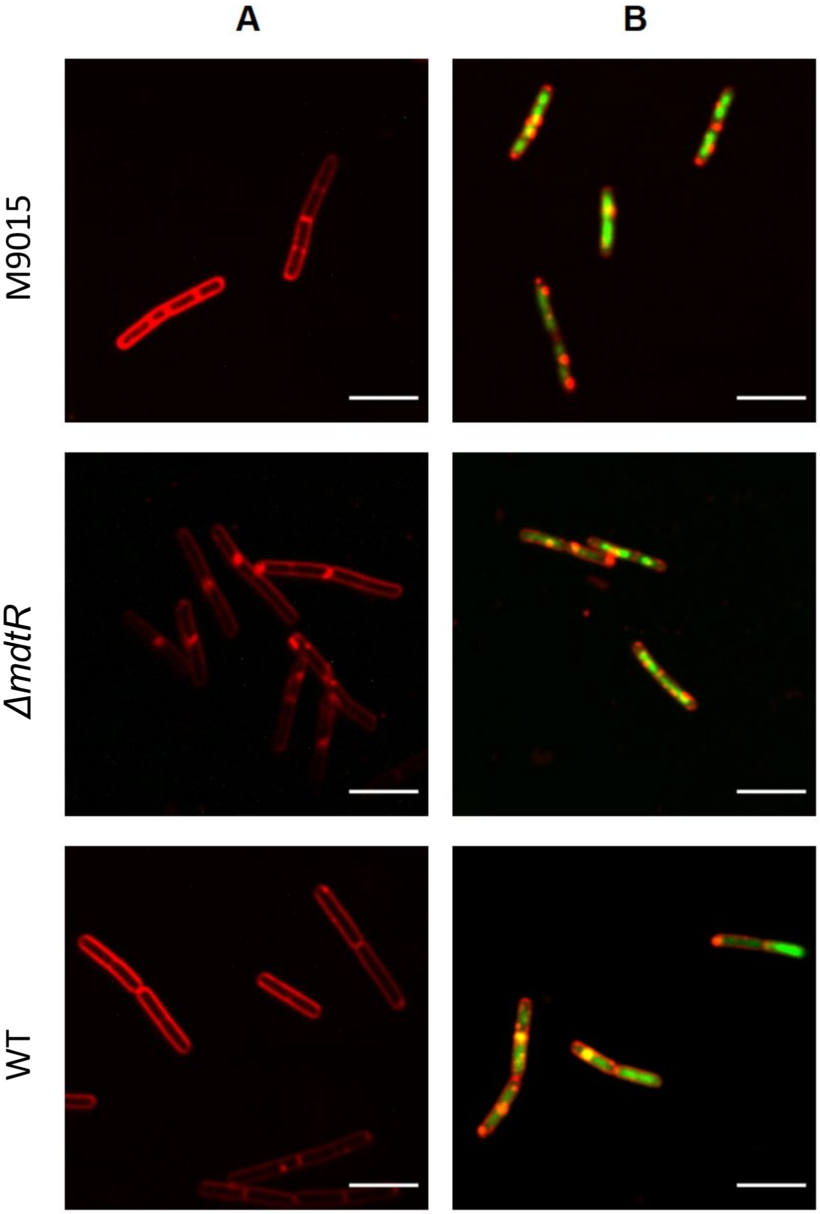

Supplementary Figure 6. Cell permeability determination of *B. Subtilis* strains. *B. Subtilis* WT and mutants were stained with SYTOX-Green (green, nucleoid, cell-impermeable) and FM4-64 (red, cell membrane), treated with DMSO (A, control) or 100 μg ml^{-1} HA (B) for 15 min, and imaged. Representative cells are shown. Scale bar is 5 μm.

1.2 Supplementary Tables

Table S1: Assignments NMR-shifts, HMBC and COSY couplings for harzianic acid (in $CDCl_3$)

	**Harzianic acid ($CDCl_3$)**			
**#**	**δC[a]**	**δH[b]**	**HMBC[b]**	**COSY[b]**
1	13.7	0.95 (t), 3H	2, 3	2
2	21.8	1.50 (m), 2H	1, 3, 4	1, 3
3	35.5	2.24 (dd), 2H	1, 2, 4, 5	3, 4 or 5[c]
4	149.9	6.37[c] (m), 1H	2, 3, 5, 6, 7[c]	3, 6[c]
5	129.6	6.38[c] (m), 1H	2, 3, 6, 7[c]	3, 6[c]
6	147.6	7.55 (m), 1H	4, 5, 7, 8	4 or 5[c], 7
7	119.1	7.00 (d, *J*=15.1 Hz), 1H	5, 6, 8, 10	6
8	176.7	-	-	-
9	173.2	-	-	-
10	99.7	-	-	-
11	197.3	-	-	-
12	64.1	3.63 (dd, *J*= 10.6, 1,0 Hz), 2H	9, 11, 13, 14, 18,	13
13	33.8	1.89 (dd), 1H	11, 12, 14, 15, 19	12
		2.48 (d), 1H		
14	79.9	-	-	-
15	36.0	2.02 (m), 1H	13, 14, 16, 17, 19	16 or 17[c]
16	17.5	0.99[c] (m), 3H	14, 15[c]	15
17	16.2	0.99[c] (m), 3H	14, 15[c]	15
18	26.6	2.97 (s), 3H	9, 12	-
19	176.3	-	-	-

[a]= measured at 150 MHz, [b]= measured at 600 MHz, [c]= overlapping signals

Table S2. HA MIC on pathogenic bacteria

MICs of HA on different pathogenic bacteria were tested starting at a 400 μg ml^{-1} which was then serially diluted with a factor 2.

Strain	Gram	MIC (μg ml^{-1})
Acinetobacter baumannii 1179 [a]	-	> 400
Acinetobacter nosocomialis 14-8211 [a]	-	> 400
Enterobacter cloacae complex MC04842 [a]	-	> 400
Escherichia coli TEM-3 GVJS004 [a]	-	> 400
Klebsiella pneumoniae SHV-18 GVJS006 [a]	-	> 400
Pseudomonas aeruginosa ATCC57853 [b]	-	> 400
Stenotrophomonas maltophilia GV20A226 [a]	-	> 400
Enterococcus faecium VRE GV16D030 [a]	+	100
Enterococcus faecium GV15A623 [a]	+	50
Listeria monocytogenes GV21-4a [a]	+	25
Staphylococcus aureus MSSA 476 GVS0101 [a]	+	50
Staphylococcus aureus MRSA USA300 GVS1474 [a]	+	200
Staphylococcus epidermidis GV08A1071 [a]	+	50
Streptococcus pneumoniae 05A396 [a]	+	25

[a] Gift from University Medical Center Utrecht;

[b] ATCC strains.

Table S3. ***B. subtilis*** **strain M9015 harbors five mutations in four genes**

Bioinformatic analysis of the genome sequences of *B. subtilis* strain 168 and M9015 results in 10 possible mutations, five of which are reliable. The position of the mutations is indicated and is based on the reference genome NC_000964.3. The mutations were visualized using Integrated Genomics Viewer and the gene names of the verified reliable mutations are indicated.

# Mutation	Position	WT	M9015	Reliable?	Mutated gene
1	353056	AAGCAGCTGATC GAGCAGCTGA	AAGCAGCTGA	No	/
2	1872540	T	C	Yes	*ymaB*
3	2152047	A	C	No	/
4	2480653	CT	C	No	/
5	2480666	GT	G	No	/
6	2581726	GTTTTTT	GTTTTTTT	No	/
7	3374690	G	GA	Yes	*mdtR*
8	3374945	AGAGGAAACGG A	AGAGGAAACGGAGG AAACGGA	Yes	*mdtR*
9	3638128	ATTTTTTTT	ATTTTTTT	Yes	*flgL*
10	3786681	G	A	Yes	*atpE*

Table S4. Exogenous Lipid II and its precursors did not affect HA antimicrobial activity

Vancomycin, nisin and HA have an antimicrobial effect on *B. subtilis*. Quenching of this antimicrobial effect was tested by addition of exogenous Lipid II and precursors (10 µM for nisin and vancomycin and either 10 µM or 150 µM for HA; results of both lipid concentrations on antimicrobial effect of HA were similar) prior to the addition of indicated antimicrobials. Bacterial growth was assessed and the assay scored as unaffected (-) or affected (+) by the addition of exogenous lipid.

Antagonist	Blank	Lipid I	Lipid II	C55-P
**Vancomycin (4 × MIC)**	-	+	+	-
**Vancomycin (2 × MIC)**	-	+	+	-
**Nisin (4 × MIC)**	-	+	+	+
**Nisin (2 × MIC)**	-	+	+	+
**HA (2 × MIC)**	-	-	-	-
**HA (1 × MIC)**	-	-	-	-

Table S5. MICs of HA on *B. subtilis* and mutants.

MICs were determined by serial dilution of HA using the indicated strain. The result of biological triplicates is depicted here. Strains were obtained from Bacillus Genetic Stock Center (BGSC).

Strain	BGSC ID	MIC to HA (μg ml^{-1})
Bacillus subtilis strain Δ*alsS*	BKK36010	50
Bacillus subtilis strain Δ*alsD*	BKK36000	50
Bacillus subtilis strain Δ*alsR*	BKK36020	50
Bacillus subtilis strain Δ*ilvB*	BKK28310	50
Bacillus subtilis strain Δ*ydaP*	BKK04340	50
Bacillus subtilis strain Δ*ilvH*	BKK28300	50

2 Supplementary Movies

Supplementary Movie 1. DBMI of cells upon DMSO treatments. *B. subtilis* cells were treated with 1% DMSO (control). Cells were stained with FM4-64 (red, cell membrane) and SYTO-9 (green, nucleoid) and imaged by time lapse confocal fluorescence microscopy for 30 min with 3 min intervals. Representative cells are shown. Scale bar is 5 µm.

Supplementary Movie 2. DBMI of cells upon HA treatments. *B. subtilis* cells were treated with HA (2.5 × MIC). Cells were stained and imaged as in Movie S1. Representative cells are shown. Scale bar is 5 µm.

Supplementary Movie 3. DBMI of cells upon nisin treatments. *B. subtilis* cells were treated with nisin (2.5 × MIC). Cells were stained and imaged as in Movie S1. Representative cells are shown. Scale bar is 5 µm.
